# Induction FOLFIRINOX followed by stereotactic body radiation therapy in locally advanced pancreatic cancer

**DOI:** 10.3389/fonc.2022.1050070

**Published:** 2022-12-14

**Authors:** Jae Hyup Jung, Changhoon Song, In Ho Jung, Jinwoo Ahn, Bomi Kim, Kwangrok Jung, Jong-Chan Lee, Jaihwan Kim, Jin-Hyeok Hwang

**Affiliations:** ^1^ Division of Gastroenterology, Department of Internal Medicine, Seoul National University Bundang Hospital, Seoul National University College of Medicine, Seongnam, Republic of Korea; ^2^ Department of Radiation Oncology, Seoul National University Bundang Hospital, Seoul National University College of Medicine, Seongnam, Republic of Korea

**Keywords:** pancreatic cancer, locally advanced pancreatic cancer, FOLFIRINOX, stereotactic body radiation therapy (SBRT), conversion surgery, prognosis

## Abstract

**Introduction:**

FOLFIRINOX (the combination of 5-fluorouracil, leucovorin, irinotecan, and oxaliplatin) is the preferred systemic regimen for locally advanced pancreatic cancer (LAPC). Furthermore, stereotactic body radiation therapy (SBRT) is a promising treatment option for achieving local control in these patients. However, clinical outcomes in patients with LAPC treated using FOLFIRINOX followed by SBRT have not been clarified. Therefore, we aimed to evaluate clinical outcomes of induction FOLFIRINOX treatment followed by SBRT in patients with LAPC.

**Methods:**

To this end, we retrospectively reviewed the medical records of patients with LAPC treated with induction FOLFIRINOX followed by SBRT in a single tertiary hospital. We evaluated overall survival (OS), progression-free survival (PFS), resection rate, SBRT-related adverse events, and prognostic factors affecting survival.

**Results:**

Fifty patients were treated with induction FOLFIRINOX for a median of 8 cycles (range: 3–28), which was followed by SBRT. The median OS and PFS were 26.4 (95% confidence interval [CI]: 22.4–30.3) and 16.7 months (95% CI: 13.0–20.3), respectively. Nine patients underwent conversion surgery (eight achieved R0) and showed better OS than those who did not (not reached vs. 24.1 months, p = 0.022). During a follow-up period of 23.6 months, three cases of grade 3 gastrointestinal bleeding at the pseudoaneurysm site were noted, which were managed successfully. Analysis of the factors affecting clinical outcomes revealed that a high radiation dose (≥ 35 Gy) resulted in a higher rate of conversion surgery (25% [8/32] vs. 5.6% [1/18], respectively) and was an independent favorable prognostic factor for OS in the adjusted analysis (hazard ratio: 2.024, 95% CI: 1.042–3.930, p = 0.037).

**Conclusion:**

Our findings suggest that induction FOLFIRINOX followed by SBRT in patients with LAPC results in better survival with manageable toxicities. A high total SBRT dose was associated with a high rate of conversion surgery and could afford better survival.

## Introduction

Pancreatic cancer (PC) is the third leading cause of cancer-related deaths in the United States and it has been responsible for 49,830 deaths thus far in 2022. The death rate for PC has increased slightly since the mid-2000s (1, 2). Surgical resection is the only curative treatment for PC; however, only 10-15% of affected patients are considered suitable for surgical resection at the time of diagnosis. Approximately 30–35% of patients were diagnosed with locally advanced PC (LAPC), and the 5-year survival rate in LAPC was less than 15% (3). Conventionally, systemic chemotherapy with or without traditional fractionated external-beam radiotherapy (EBRT) was considered the standard of treatment for patients with LAPC (4–6). However, some randomized controlled trials (7, 8) investigating EBRT have reported unsatisfactory results in terms of efficacy, with considerable radiation-related adverse events (AEs). Moreover, conventional EBRT with concurrent chemotherapy may require quite a few weeks for completion (9).

Since the publication of a randomized trial by Conroy et al. in 2011 (10), the combination of folinic acid, fluorouracil, irinotecan, and oxaliplatin (FOLFIRINOX) has become the standard of care for metastatic PC (11). Several studies have demonstrated that FOLFIRINOX is also effective in LAPC; thus, FOLFIRINOX is the preferred systemic chemotherapy regimen in patients with good performance status (12, 13). Furthermore, the 2022 National Comprehensive Cancer Network guidelines recommend FOLFIRINOX as the preferred systemic treatment for patients with LAPC (14).

However, over 70% of patients with LAPC are ineligible candidates for resection even after induction chemotherapy because either their lesions are not sufficiently reduced in size to be suitable for surgery or due to locoregional progression (15–18). Therefore, local ablative therapies have been explored as new therapeutic options for patients with LAPC, which could increase locoregional disease control rates (19–21). Among them, stereotactic body radiation therapy (SBRT) is a promising treatment that can overcome radio-resistance because it allows precise delivery of high-dose radiation while reducing radiation treatment-related AEs. The latter is achieved by decreasing the radiation dose delivered to adjacent healthy tissue compared to that associated with conventional EBRT (6, 22, 23).

In 2004, Koong et al. (24) conducted a dose-escalation study using SBRT for pancreatic cancer, which showed favorable results in terms of local disease control. In several retrospective (25–29) and single-arm prospective studies (LAPC-1 trial) (30, 31), sequential treatment with induction chemotherapy (FOLFIRINOX or other regimens) followed by SBRT yielded encouraging results in terms of local control in patients with LAPC. Moreover, SBRT was associated with a favorable rate of conversion surgery among patients with LAPC, which could result in better survival (32).

The addition of SBRT is a promising treatment option for patients with LAPC; however, no consensus exists regarding the patients suitable for this treatment, when it should be administered, and the clinical factors that should be considered for better clinical outcomes (14, 33, 34). Therefore, in the present study, we aimed to evaluate clinical outcomes of induction FOLFIRINOX followed by SBRT in patients with LAPC at a single tertiary teaching hospital.

## Patients and methods

### Study patients

Electronic medical records of patients with LAPC who were treated between December 2015 and September 2020 at a single tertiary teaching hospital (Seoul National University Bundang Hospital, Seoungnam, South Korea) were retrospectively reviewed. The patients were treated with induction FOLFIRINOX regimen (oxaliplatin, 85 mg per m^2^ of the body-surface area; irinotecan, 180 mg per m^2^; leucovorin, 400 mg per m^2^; and fluorouracil, 400 mg per m^2^ delivered as a bolus followed by 2400 mg per square meter administered as a 46-hour continuous infusion, every 2 weeks) followed by SBRT (11). The inclusion criteria were as follows: (1) patients with LAPC diagnosed based on the results of radiological evaluations and a multidisciplinary conference, (2) patients who had received induction FOLFIRINOX (≥1 cycle) and were unsuitable candidates for conversion surgery despite induction FOLFIRINOX based on multidisciplinary discussion, (3) patients who revealed no evidence of metastatic disease or gastric or duodenal invasion at the time of SBRT, (4) patients who had not previously received abdominal radiotherapy, and (5) patients without a history of other malignancies within 5 years.

### Study design and definition of clinical outcomes

The patients’ baseline characteristics were assessed at diagnosis and before SBRT. Overall survival (OS), progression-free survival (PFS), resection rate, SBRT-related AEs, and prognostic factors were assessed. Furthermore, survival, disease progression, and resection data until 31 March 2022 were evaluated. OS was defined as the time from histological diagnosis to all-cause death or the last follow-up. PFS was defined as the time from histological diagnosis to radiological progression according to the Response Evaluation Criteria in Solid Tumors criteria version 1.1, all-cause death, or last follow-up. Locoregional progression was defined as disease progression within the primary tumor or peripancreatic lymph nodes, and distant progression was defined as distant metastasis. For those who underwent conversion surgery, T and N stages were assessed using resected specimens according to the eighth edition of the American Joint Committee on Cancer Staging System. The pathological response of the tumor to previous chemotherapy or radiotherapy was assessed according to the tumor regression scoring system of the College of American Pathologists (CAP) version 4.2. SBRT-related AEs were assessed according to the National Cancer Institute Common Terminology for Adverse Events version 5.0. SBRT-related acute and late AEs were defined as AEs occurring within 90 days and after 90 days from radiation therapy, respectively.

### SBRT procedure

Patients were treated with five-fraction SBRT on 5 consecutive business days by using a Varian TruBeam linear accelerator (Varian Medical Systems Inc., Palo Alto, CA, USA). SBRT was initiated within 2 weeks after the completion of chemotherapy. At the time of simulation, a four-dimensional computed tomography (CT) (Philips Brilliance Big Bore CT scanner, Philips Medical Systems, Cleveland, OH, USA) simulation was performed during free breathing to determine the position variation of the pancreas and organ at risk (OAR). The respiratory cycle was recorded using an abdominal bellows strap (Philips Healthcare, Best, Netherlands). Thin-sliced CT scans with intravenous contrast were obtained, with patients positioned supine and arms above the head in a Body Pro-Lok ONE device (CIVCO Medical Solutions, Orange City, IA, USA) for immobilization. Pre-treatment diagnostic CT or magnetic resonance imaging (MRI) scans were matched if they provided better delineation of the tumor than did simulation CT images.

The Eclipse planning system was used for target and OAR delineation and treatment planning. Gross tumor volume (GTV) included the gross tumor and adjacent vessels, such as the common hepatic artery, celiac axis, and/or superior mesenteric vessels. The internal target volume (ITV) was obtained by summing the GTVs for all respiratory phases. The planning target volume (PTV) was generated by adding a 2-mm margin circumferentially and a 4- to 6-mm margin craniocaudally to the ITV. A 3-mm margin was added to the OAR volumes to obtain the planning OAR volume (PRV). The modified PTV was obtained from the PTV by subtracting the PRV. The desired prescribed dose was 40 Gy delivered in five fractions. Ninety-five percent of the modified PTV should be covered by the prescribed dose and at least 95% of the PTV should be covered by 30 Gy. If the desired prescribed dose violated the constraints of the OARs, the prescription dose was lowered from 40 to 35, 33, or 30 Gy. The OAR constraints were as follows: stomach and duodenum: Dmax ≤ 32 Gy, V20 < 3 cc, and V15 < 9 cc, and other small bowel intestine: Dmax: 35 Gy and V20 < 20 cc. Cone-beam CT was performed for positional validation before the delivery of each fraction. Daily cone beam CT 3-dimensional images without fiducial were registered to planning CT images. Patients were aligned to the spine and then shifted to align to great vessels, including the aorta, celiac axis, and/or superior mesenteric artery. Although the soft tissue is rarely visible on cone beam CT, soft tissue was sometimes used in alignment when visible.

### Statistical analysis

To compare the patients’ baseline characteristics, chi-square or Fisher’s exact test was used for categorical scales, and the t-test or Mann-Whitney U test was used for numerical scales. OS and PFS were evaluated using the Kaplan-Meier method, and differences in survival were analyzed using the log-rank test. The Cox proportional hazards model was used to analyze survival and other factors. The values of all continuous variables were dichotomized on the entire sample (< median vs. ≥ median) in univariate and multivariate cox proportional analyses. All tests were double-sided with a p-value of less than 0.05 being statistically significant. Multivariate analysis was performed using variables with p-values of less than 0.1 in the univariate analyses. All statistical analyses were performed using SPSS software version 25 (IBM Corporation, New York, USA) and R software version 4.2.0 (R Foundation for Statistical Computing, Vienna, Austria).

## Results

### Patient characteristics

Fifty patients were retrospectively evaluated, and the median follow-up period was 23.6 months. Among them, 39 (78.0%) patients died during the follow-up period and 11 (22.0%) were alive until March 31, 2022. The median age of the patients was 64.1 (range: 47.8–81.6) years. Twenty-eight (56.0%) patients were female, and 30 (60.0%) had pancreatic head or neck cancer. The median body mass index was 22.7 kg/m^2^ at diagnosis and before SBRT. The median serum albumin and CA 19-9 levels changed from 4.0 to 3.9 g/dL and from 106.0 to 48.5 U/mL, respectively, after induction FOLFIRINOX. The median tumor size changed from 3.2 to 2.9 cm after induction FOLFIRINOX. The median number of cycles and duration of FOLFIRINOX treatment was 8 (range: 3–28) and 4.9 (range: 1.4–21.7) months, respectively. Thirty-nine (78.0%) patients showed stable disease as the best response during induction FOLFIRINOX, while 11 (22.0%) showed partial response. The median time to SBRT from diagnosis, the total dose of SBRT, and SBRT dose per fraction was 6.1 (range: 2.8–22.3) months, 35 (range: 30–40) Gy in five fractions, and 7 (6–8) Gy, respectively. Nine (18.0%) patients underwent conversion surgery after SBRT during the follow-up period (median: 3.5 months, range: 0.8–11.7 months) (**Table 1**).

**Table 1:** Baseline characteristics.

Characteristics	Statistical value
Age (yr), median	64.1 (47.8–81.6)
Sex	
Female	28 (56.0)
Male	22 (44.0)
Primary site	
Head or Neck	30 (40.0)
Body or Tail	20 (60.0)
At diagnosis	
BMI (kg/m^2^), median	22.7 (17.5–26.1)
Serum albumin (g/dL), median	4.0 (2.7–4.8)
CA 19-9 (U/mL), median	106.0 (2–7999)
Tumor size (cm), median	3.2 (1.9–8.3)
Pre-SBRT	
BMI (kg/m^2^), median	22.7 (16.5–27.6)
Serum albumin (g/dL), median	3.9 (2.5–4.7)
CA 19-9 (U/mL), median	48.5 (5–1780)
Tumor size (cm), median	2.9 (1.4–5.7)
Induction FOLFIRINOX cycles, median	8.0 (3–28)
Induction FOLFIRINOX duration (months), median	4.9 (1.4–21.7)
Best response during induction FOLFIRINOX	
SD	39 (78.0)
PR	11 (22.0)
Time to SBRT from diagnosis (months), median	6.1 (2.8–22.3)
Total SBRT dose (Gy), median	35 (30–40)
SBRT dose per fraction (Gy), median	7 (6–8)
Conversion surgery	
Yes	9 (18.0)
No	41 (82.0)
Time to conversion surgery from SBRT (months), median	3.5 (0.8–11.7)

Data are presented as median (range) or No. of patients/total no. (n%), unless otherwise stated; BMI, body mass index; CA 19-9, carbohydrate antigen 19-9; SD, stable disease; PR, partial response; SBRT, stereotatic body radiation therapy; Gy, gray.

### Efficacy

**Figure 1:**
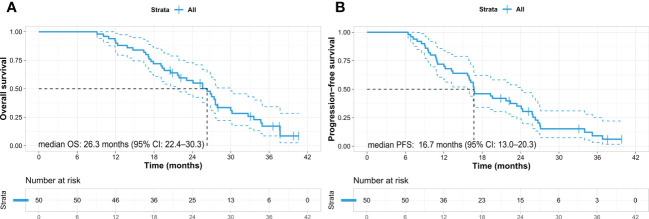
Kaplan–Meier survival curves in the entire cohort of pancreatic cancer patients. **(A)** Overall survival and **(B)** progression-free survival. OS, overall survival; PFS, progression-free survival; CI, confidence interval.

**Figure 2:**
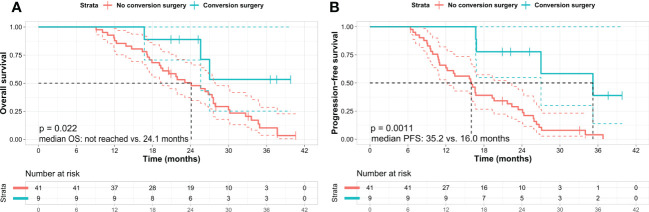
Kaplan–Meier survival curves in patients according to the conversion surgery. **(A)** Overall survival and **(B)** progression-free survival. Kaplan–Meier analysis shows that the patients who underwent conversion surgery exhibit better OS and PFS than those who did not. Log-rank test *p*-value was **(A)** 0.022 and **(B)** 0.001 between the two subgroups, respectively. OS, overall survival; PFS, progression-free survival.

The patients’ median OS and PFS were 26.3 months (95% confidence interval [CI]: 22.4–30.3) and 16.7 months (95% CI: 13.0–20.3), respectively (**Figures 1A, B**). Nine patients (18%) who underwent conversion surgery showed longer OS than did those who did not (not reached vs. 24.1 months, *p* = 0.022). (**Figure 2A**) and longer median PFS (35.2 months vs. 16.0 months, *p* = 0.001) (**Figure 2B**). Among them, eight underwent margin-negative resection. The T and N stage distributions were as follows: five in T1 and four in T2 and seven in N0 and two in N1. One patient revealed a near-complete response (CAP grade 1), and eight exhibited a partial response (CAP grade 2) (**Table 2**). Among the 34 patients who exhibited disease progression after SBRT, 9 (26.5%) showed locoregional progression without distant metastasis (**Table 3**).

**Table 2:** Pathological and clinical characteristics of patients who underwent conversion surgery.

Pathology	No. of patients
Total patients	9
Resection margin	
R0	8
R1	1
T stage	
T1	5
T2	4
N stage	
N0	7
N1	2
Response to previous treatment	
Grade 0 (complete response)	0
Grade 1 (near complete response)	1
Grade 2 (partial response)	8
Grade 3 (poor or no response)	0
Death within 6 months postoperatively^a^	1

T stage and N stage were assessed using American Joint Committee on Cancer staging system 8^th^; Response of tumor to previous CT or RT was assessed using tumor regression scoring system in College of American Pathologists [version 4.2]. ^a^One patient died of bowel perforation, a surgical complication.

**Table 3:** Pattern of disease progression.

Category	No. of patients (%)
Disease progression	34 (68)
Locoregional progression only	9
Distant progression	23
Liver	12
Peritoneal seeding	7
Lung	2
Distant lymph node	1
Multiple sites	3
No disease progression ^a^	16 (32)

Data are presented as the no. of patients/total no. (n%). ^a^Three patients who died without radiological evidence of disease progression due to liver abscess, gastric variceal bleeding, and bowel perforation were included.

### SBRT-related acute and late AEs

SBRT-related AEs of grade 3 or higher occurred within 1 year of SBRT in three patients who had gastrointestinal (GI) bleeding at the pseudoaneurysm site. These bleeding events were controlled by supportive management and transarterial embolization (dose ≥ 35 Gy in two patients and < 35 Gy in one). SBRT-related acute AEs of grade 2 or lower included anorexia (three patients), fatigue (three patients), nausea (three patients), vomiting (one patient), and diarrhea (five patients). SBRT-related late AEs of grade 2 or lower were gastritis (two patients), GI ulcer (four patients), and non-significant GI bleeding (one patient). These complications were well-managed conservatively, and there were no deaths due to these complications (**Table 4**).

**Table 4:** Radiation treatment-related acute and late adverse events.

Category	Grade 1	Grade 2	Grade 3
Acute adverse effects			
Anorexia	2	1	0
Fatigue	2	1	0
Nausea	2	1	0
Vomiting	1	0	0
Diarrhea	1	4	0
Late adverse effects			
Gastritis	0	2	0
GI ulcer	0	4	0
GI bleeding	0	1^a^	3^b^

Adverse events are assessed using the National Cancer Institute Common Terminology for Adverse Events [version 5.0].; Acute adverse events denote adverse events within 90 days from radiation therapy; Late adverse events denote adverse events after 90 days from radiation therapy.; ^a^Gastric ulcer bleeding (1 in ≥ 35 Gy), ^b^Three pseudoaneurysm site bleeding (2 in ≥ 35 Gy, 1 in < 35 Gy).

### Clinical factors affecting survival

**Figure 3:**
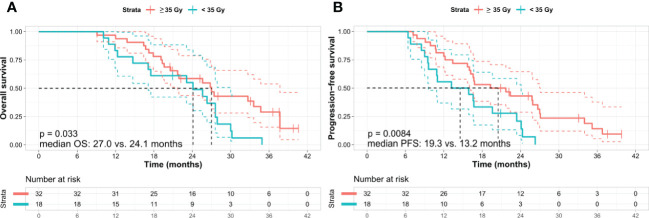
Kaplan–Meier survival curves according to the total dose of SBRT. **(A)** Overall survival and **(B)** progression-free survival. Kaplan–Meier analysis shows that the patients treated with a high total dose of SBRT (≥ 35 Gy in five fractions) exhibit better OS and PFS than those who received a low total dose of SBRT (< 35 Gy in five fractions). Log-rank test *p*-values were **(A)** 0.033 and **(B)** 0.008 between the two subgroups, respectively. OS, overall survival; PFS, progression-free survival; Gy, gray.

The two variables (Tumor size (pre-SBRT) and Total SBRT dose) with a p-value of less than 0.1 in univariate cox analysis were used for adjusted analysis in OS and PFS. Analysis of OS by using adjusted variables showed that a high total dose of SBRT was an independent and significant favorable prognostic factor (≥ 35 vs. < 35 Gy; 27.0 vs. 24.1 months; hazard ratio [HR] 2.024; 95% CI 1.042–3.930; p = 0.037) (**Table 5**, **Figure 3A**), although there were no statistically significant differences between the high and low total SBRT dose groups in terms of baseline characteristics (**Supplementary Table**). Moreover, a higher total dose of SBRT resulted in a higher resection rate than did a lower total dose of SBRT (25.0% vs. 5.6%, p = 0.086). Analysis of PFS using adjusted variables showed that a high total dose of SBRT (≥ 35 vs. < 35 Gy; 19.3 vs. 13.2 months; HR 2.364; 95% CI 1.218–4.588, p = 0.011) and small tumor size (< 2.9 vs. ≥ 2.9 cm; 23.4 vs. 15.9 months; HR: 1.853; 95% CI: 1.005–3.416, p = 0.048) were independent and significant favorable prognostic factors (**Table 6** and **Figure 3B**).

**Table 5:** Univariate and multivariate analyses of the overall survival at diagnosis.

Variables	No. of patients	OS (median, months)	95% CI	Univariate analysis			Multivariate analysis		
				HR	95% CI	*p* value	HR	95% CI	*p* value
Total patients	50	26.4	22.4–30.3						
Age (years)									
< 65	29	26.8	17.2–36.5						
≥ 65	21	25.6	21.0–30.2	1.044	0.568–1.918	*0.891*			
Sex									
Male	22	27.4	18.9–35.9						
Female	28	25.6	22.2–29.0	1.269	0.663–2.430	*0.472*			
Primary site									
Body and Tail	20	27.6	25.6–29.6						
Head and Neck	30	23.1	15.2–31.0	1.554	0.806–2.996	*0.189*			
CA 19-9 (Pre-SBRT)									
< 48.5 U/mL	25	26.8	24.6–29.1						
≥ 48.5 U/mL	25	24.1	18.9–29.2	1.187	0.626–2.253	*0.599*			
Tumor size (cm) (Pre-SBRT)									
< 2.9	25	27.0	24.5–29.6						
≥ 2.9	25	21.7	15.1–28.2	1.722	0.914–3.244	*0.093*	1.723	0.914–3.249	*0.093*
Induction FOLFIRINOX cycles									
≥ 8	34	25.6	22.0–29.2						
< 8	16	26.8	19.6–34.1	0.950	0.485–1.859	*0.880*			
Best response									
PR	11	25.6	13.9–37.3						
SD	39	26.4	21.7–31.0	1.007	0.693–1.465	*0.969*			
Total SBRT dose									
≥ 35 Gy	32	27.0	20.7–33.4						
< 35 Gy	18	24.1	19.3–28.9	2.017	1.043–3.901	*0.037*	2.024	1.042–3.930	*0.037*

OS, overall survival; CI, coefficient index; HR, hazard ratio; SBRT, stereotactic body radiation therapy; CA 19-9, carbohydrate antigen 19-9; PR, partial response; SD, stable disease; Gy, gray.

**Table 6:** Univariate and multivariate analyses of the progression-free survival at diagnosis.

Variables	No. of patients	PFS (median, months)	95% CI (months)	Univariate analysis			Multivariate analysis		
				HR	95% CI	*p* value	HR	95% CI	*p* value
Total patients	50	16.7	13.0–20.3						
Age (years)									
< 65	29	16.7	11.9–21.6						
≥ 65	21	16.6	15.3–18.0	1.044	0.568–1.918	*0.891*			
Sex									
Female	28	16.7	8.9–24.6						
Male	22	16.6	15.4–17.7	1.227	0.667–2.257	*0.510*			
Primary site									
Body and Tail	20	16.6	16.4–16.8						
Head and Neck	30	16.7	7.2–26.2	0.986	0.532–1.857	*0.986*			
CA 19-9 (Pre-SBRT)									
< 48.5 U/mL	25	19.3	8.2–30.4						
≥ 48.5 U/mL	25	16.6	15.4–17.7	1.330	0.724–2.442	*0.358*			
Tumor size (Pre-SBRT)									
< 2.9 cm	25	23.4	11.7–35.1						
≥ 2.9 cm	25	15.9	7.9–24.0	1.860	1.012–3.418	*0.046*	1.853	1.005–3.416	*0.048*
Induction FOLFIRINOX cycles									
≥ 8	34	16.7	12.7–20.6						
< 8	16	16.0	14.0–17.9	1.124	0.597–2.117	*0.716*			
Best response									
PR	11	16.7	13.0–20.4						
SD	39	16.7	12.4–20.9	0.936	0.647–1.354	*0.725*			
Total SBRT dose									
≥ 35 Gy	32	19.3	11.8–26.8						
< 35 Gy	18	13.2	3.0–23.4	2.369	1.226–4.580	*0.010*	2.364	1.218–4.588	*0.011*

PFS, progression free survival; CI, coefficient index; HR, hazard ratio; SBRT, stereotactic body radiation therapy; CA 19-9, carbohydrate antigen 19-9; PR, partial response; SD, stable disease; Gy, gray.

## Discussion

In the present study, we investigated the feasibility of induction FOLFIRINOX followed by SBRT as a strategy for local control and increased possibility of conversion surgery in patients with LAPC. Furthermore, we explored whether this strategy improves survival. After discussing multidisciplinary approach for one patient whose disease was stable but remained unresectable after sufficient FOLFIRINOX, sequential SBRT was conducted on patients who considered it helpful. Resultantly, 18.0% of patients who were considered unsuitable candidates for surgery despite induction FOLFIRINOX could undergo conversion surgery, and most patients achieved R0 resection. Moreover, the first recurrency occurred more often at the distant site than at the locoregional site, and SBRT-related AEs were rare and manageable. Therefore, induction FOLFIRINOX followed by SBRT may be a promising treatment strategy for patients who remained unresectable despite induction FOLFIRINOX, given its considerable efficacy (conversion rate and locoregional control rate) and acceptable SBRT-related AEs.

Several recent studies have investigated the issue of induction chemotherapy followed by SBRT. Mellon EA et al. (27) studied 49 patients with LAPC who received induction chemotherapy (43% of them were treated with FOLFIRINOX) followed by SBRT (30 Gy in five fractions), and their results showed a median OS of 15 months. Moningi S et al. (28) also reported similar results in 74 patients with LAPC who received induction chemotherapy (24% of them were treated with FOLFIRINOX) followed by SBRT (25–33 Gy in five fractions), with a median OS of 18.4 months. These results were worse than our results (median OS of 26.3 months), probably due to the reduced dose of SBRT and lower potency of induction chemotherapy. This suggestion is supported by a previous study (29) in which a combination of modified FOLFIRINOX and a higher SBRT dose (≥ 40 Gy in five fractions) reported results similar to ours (median OS of 24 months). Conversely, a small prospective trial (LAPC-1 trial) (30, 31) reported an OS of 18 months in 39 patients treated with induction FOLFIRINOX followed by SBRT (40 Gy in five fractions), which was worse than the OS (26 months) we identified.

Not all patients in the present study could undergo resection despite prior induction FOLFIRINOX. However, after additional SBRT, nine patients (18%) could undergo curative resection (R0 resection in eight and N0 in seven); this finding was similar to that shown in other studies (27, 28, 30, 31). However, results of the present study cannot be explained solely based on SBRT because of selection bias due to the retrospective nature of the study. Nevertheless, considering that 18% of the patients who were unsuitable candidates for surgery after sufficient induction chemotherapy (median eight cycles of FOLFIRINOX) were able to undergo resection after continuing FOLFIRINOX with simultaneous SBRT, SBRT may arguably play a role in these patients. Recently, in a phase 2 randomized clinical trial (35), neoadjuvant FOLFIRINOX was used in patients with borderline resectable PC with or without hypofractionated radiation therapy. That trial showed that additional hypofractionated radiation therapy did not improve the 18-month OS and R0 resection rates. However, 12.5% of the patients in the study received a lower radiation dose (hypofractionated image-guided radiotherapy: 25 Gy in five fractions), which could have influenced the outcomes, since a higher radiation dose was associated with better outcomes in ours and other studies (29, 36).

SBRT-related AEs in the present study were well tolerated and managed, which was similar to that in previous studies (24–31). Furthermore, these AEs were less frequent than those associated with conventional EBRT (7–9). It is well known that GI bleeding is a severe late complication in patients and is more often observed in those who receive single-fraction SBRT compared with that in those who receive multi-fraction SBRT (37). In the present study, in which all patients received five fractions, three cases of grade 3 GI bleeding at the pseudoaneurysm site were noted and were well controlled by transarterial embolization, which was similar to the results of other studies (29, 30). One patient who died due to bowel perforation occurred sequentially superior mesenteric artery and superior mesenteric vein thrombosis, bowel infarction, and bowel perforation within five months after surgery. SBRT could contribute to the increased difficulty of surgery that resulted in severe surgical complications. Still, it is difficult to determine a direct causality and cannot be explained solely based on SBRT.

A higher total dose of SBRT (35 Gy) showed a trend toward better OS than did a dose of 30 Gy or less. This finding was similar to that in other studies (29, 36). Moreover, 25% (8 of 32) of the patients treated with a higher total dose of SBRT underwent surgery subsequently, compared with 5% (1 of 18) treated with a lower total dose of SBRT. However, the two groups were not significantly different in terms of radiation-related AEs. Taken together, these findings suggest that when additional SBRT is necessary and feasible for LAPC, a higher total dose of SBRT may be recommended, considering its efficacy and safety. More prospective studies are needed to determine the appropriate SBRT protocols and whether they have clinical benefits.

This study has some limitations. First, this was a retrospective study conducted in a single tertiary center, which may have resulted in selective bias. However, the enrolled patients had a uniform disease status and remained unsuitable candidates for resection after induction FOLFIRINOX and received radiation therapy using a uniform SBRT protocol, which provided informative results that were easy to apply in clinical practice. Second, we did not use fiducial marker placement to target tumors accurately during SBRT because this product was unavailable for clinical practice in Korea. However, SBRT without fiducial markers in our study was associated with manageable AEs compared with those associated with SBRT in other studies that used fiducial markers (38). Third, the interval and total cycles of FOLFIRINOX were not standardized because of this study’s retrospective design. FOLFIRINOX was continued until sequential SBRT was initiated, which was decided in a multidisciplinary discussion. However, except for one extreme case (28 cycles), the FOLFIRINOX cycles for the remaining patients ranged from 3 to 16. Moreover, the median cycle of FOLFIRINOX was similar to that used in other studies (30, 31).

The strategy of adding SBRT to LAPC patients who had received FOLFIRINOX was not standardized. LAPC-1 trial (30, 31) showed the advantage of SBRT followed by induction FOLFIRINOX in improving survival in patients initially inoperable at diagnosis. A large-sample size study (Gemenetzis et al.) (32) revealed that additional SBRT would contribute to an increased resection rate in patients with LAPC suitable for surgical exploration after FOLFIRINOX. However, there has yet to be a consensus on the role of SBRT in which clinical situations SBRT may be beneficial in LAPC patients who have received induction chemotherapy. Our study enrolled patients who remained unresectable (with reduced CA 19-9 but no significant change in tumor size) despite sufficient chemotherapy. Furthermore, among nine patients who underwent resection in our study, five patients received induction FOLFRINOX for more than eight cycles (range 10-15 cycles), unlike the LAPC-1 trial (induction FOLFIRINOX up to 8 cycles). SBRT may be helpful when curative resection is not possible despite sufficient induction chemotherapy in actual clinical practice. Our results may be valuable when making a decision (adding SBRT vs. continuing FOLFIRINOX) in the patients who remained unresectable despite sufficient chemotherapy. Furthermore, our study aimed to identify clinical factors influencing a better prognosis for these strategies and revealed that a higher total dose of SBRT could result in a better resection rate and OS.

In conclusion, induction FOLFIRINOX followed by SBRT in LAPC results in favorable OS and PFS with manageable AEs related to SBRT. A high total dose of SBRT (≥ 35 Gy in five fractions) can improve survival with a higher resection rate.

## Data availability statement

The raw data supporting the conclusions of this article will be made available by the authors, without undue reservation.

## Ethics statement

The studies involving human participants were reviewed and approved by the institutional review board of Seoul National University Bundang Hospital. Written informed consent for participation was not required for this study in accordance with the national legislation and the institutional requirements.

## Author contributions

JJ, CS, and J-HH conceived and designed research. JJ, IJ, JA, BK, and KJ collected and assembled the data. JJ and J-HH performed or supervised analyses. JJ, J-CL, JK, CS, and J-HH interpreted the results. JJ and J-HH performed statistical expertise. JJ, CS, and J-HH wrote sections of the initial draft. J-CL and JK provided substantive suggestions for revision. J-CL, JK, CS, J-HH provided the provision of study materials or patients. All authors contributed to the article and approved the submitted version.
