# Supplementary material for: Induction FOLFIRINOX followed by stereotactic body radiation therapy in locally advanced pancreatic cancer
Source: Front Oncol. 2022 Dec 14;12:1050070. doi: 10.3389/fonc.2022.1050070 (PMC9812488; doi:10.3389/fonc.2022.1050070)
Supplement: Supplementary file 1 [file Table_1.docx]

Supplementary Material

# Supplementary Figures and Tables

## Supplementary Table

**Subgroup analysis according to total dose of SBRT**

| **Characteristics** | **Higher dose (≥ 35 Gy)** | **Lower dose (< 35 Gy)** | ***P* value** |
| --- | --- | --- | --- |
| Age (yr), median | 62.9 (48.6–81.5) | 64.7 (47.8–81.6) | *0.390* |
| Sex |  |  | *0.585* |
| Female | 17 (53.1) | 11 (61.1) |  |
| Male | 15 (46.9) | 7 (38.9) |  |
| Primary site |  |  | *0.470* |
| Head or Neck | 18 (56.3) | 12 (66.7) |  |
| Body or Tail | 14 (43.8) | 6 (33.3) |  |
| At diagnosis |  |  |  |
| BMI (kg/m^2^), median | 23.1 (18.3–26.0) | 21.1 (17.5–26.1) | *0.006* |
| Serum albumin (g/dL), median | 4.0 (2.7–4.8) | 3.9 (2.7–4.8) | *0.860* |
| CA 19-9 (U/mL), median | 88.5 (2–7999) | 163.0 (4.8–4200) | *0.350* |
| Tumor size (cm), median | 3.4 (2.2–6.5) | 3.1 (1.9–8.3) | *0.746* |
| At pre-SBRT |  |  |  |
| BMI (kg/m^2^), median | 22.9 (16.7–27.6) | 21.2 (16.5–27.1) | *0.041* |
| Serum albumin (g/dL), median | 3.9 (2.5–4.7) | 4.0 (2.8–4.4) | *0.895* |
| CA 19-9 (U/mL), median | 42.5 (5–1780) | 107 (5–960) | *0.168* |
| Tumor size (cm), median | 2.9 (1.4–5.7) | 3.1 (1.4–5.4) | *0.840* |
| Induction FOLFIRINOX cycles, median | 8.0 (3–28) | 9.0 (6–12) | *0.846* |
| Induction FOLFIRINOX duration (months), median | 4.6 (1.3–21.7) | 5.3 (2.7–7.3) | *0.864* |
| Best response during induction FOLFIRINOX |  |  | *0.163* |
| SD | 23 (71.9) | 16 (88.9) |  |
| PR | 9 (28.1) | 2 (11.1) |  |
| Time to SBRT from diagnosis (months), median | 6.0 (2.8–22.3) | 6.3 (3.9–13.8) | *0.436* |
| Conversion surgery |  |  | *0.086* |
| Yes | 8 (25.0) | 1 (5.6) |  |
| No | 24 (75.0)) | 17 (94.4) |  |
| SBRT-related AEs |  |  |  |
| Gr 3 or above GI bleeding | 2 | 1 |  |
| Gr 2 GI bleeding | 0 | 1 |  |
| Gr 2 Gastric ulcer | 3 | 4 |  |
| *Data are presented as median (range) or No. of patients/total no. (n%), unless otherwise stated.; Adverse effects was assessed using the National Cancer Institute Common Terminology for Adverse Events [version 5.0].; BMI, body mass index; CA 19-9, carbohydrate antigen 19-9; SD, stable disease; PR, partial response; SBRT, stereotatic body radiation therapy; Gy, gray; AEs, adverse effects; | | | |
